# Prototype REBCO Z1 and Z2 shim coils for ultra high-field high-temperature superconducting NMR magnets

**DOI:** 10.1038/s41598-020-78644-0

**Published:** 2020-12-15

**Authors:** Dongkeun Park, Jiho Lee, Juan Bascuñán, Zhuyong Li, Yukikazu Iwasa

**Affiliations:** 1 Francis Bitter Magnet Laboratory/Plasma Science and Fusion Center, Massachusetts Institute of Technology, Cambridge, MA 02139 USA; 2 Institute for Basic Science, Daejeon, 34126 South Korea; 3 Department of Electrical Engineering, Shanghai Jiao Tong University, Shanghai, 200240 China

**Keywords:** Biomedical engineering, NMR spectroscopy

## Abstract

We present promising results of novel high-temperature superconducting (HTS) shim coil prototypes that circumvent the size and strength limitation of our earlier innovative HTS shim concept based on 46-mm wide REBCO tape. The HTS shim coil is placed inside the HTS magnet, mainly for ultra-high-field (> 1 GHz or 23.5 T) NMR magnets, and thus unaffected from the windings’ diamagnetic wall effects. One full-scale version will be applied to clean up Z1 and Z2 harmonic errors in the MIT 1.3-GHz high-resolution NMR magnet composed of an 835-MHz HTS insert, while another version for an MIT 1-GHz microcoil NMR magnet whose small-scale model we are currently building. The prototype sets were wound with a 2-pile, 1.03-mm wide, 0.30-mm thick REBCO conductor. Operated at 77 K, the Z1 shim set generated a 1st harmonic field strength of 179 kHz/cm at 70 A, while the Z2 shim set, composed of two pairs, Z2_1_ and Z2_2_, generated the 2nd harmonic field of 141 kHz/cm^2^ at 50 A. Together with discussion on technical challenges for this REBCO shim coil concept, we demonstrate its feasibility for the next generation of ultra-high-field (UHF) HTS NMR magnets.

## Introduction

**Figure 1:**
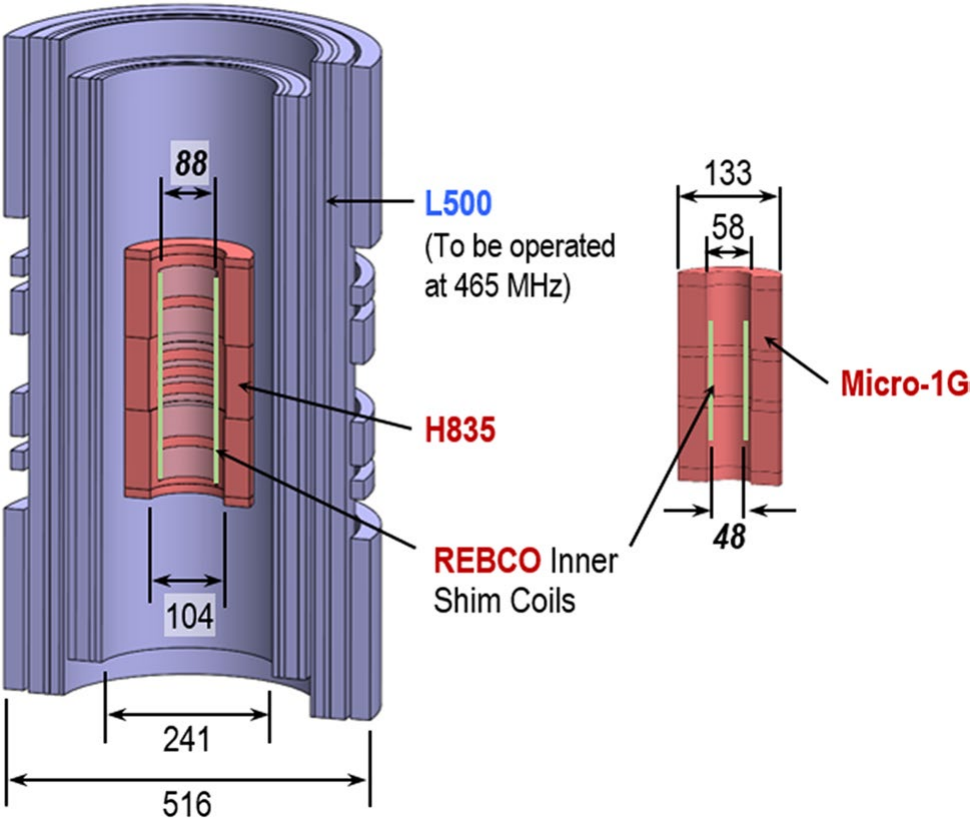
MIT 1.3-GHz HTS/LTS NMR magnet (left) and 1-GHz REBCO microcoil NMR magnet (right) with REBCO Z1, Z2 shim coils. Dimensions in mm.

A nuclear magnetic resonance (NMR) magnet requires spatially homogeneous (typically ≤ 0.01 ppm) and temporally stable (≤ 0.01 ppm/hour) field over, generally, 1-cm diameter spherical volume (DSV) at the center where the sample is placed^1^. In most as-built NMR magnets, however, nonzero tolerances in manufacturing parts, including superconductors, make the field less homogeneous than computed, i.e., field shimming is an absolutely necessity. Ultra-high-field (UHF, ≥ 1-GHz) NMR magnets composed of high-temperature superconducting (HTS) coils require shim field strengths greater than those required by < 1-GHz NMR magnets of all low-temperature superconducting (LTS) coils. Note that 23.5 T, a field corresponding to a proton frequency of 1-GHz, is the upper practical limit even when such an all-LTS magnet is operated at 1.8 K: HTS is mandatory for UHF magnets. In superconducting magnets, of all-LTS, LTS/HTS, and all HTS, superconductor magnetization generates an error field, the so-called screening-current-induced field (SCF)^2–5^, which for rare-earth barium copper oxide (REBCO) HTS, having “filament” typically of millimeters, is much greater than for LTS filament of tens of micrometers. Furthermore, a shim field from outside the magnet assembly, where the shim coils are located in conventional LTS NMR magnets, is attenuated and distorted when it penetrates through the HTS winding to reach the center, because it acts as a diamagnetic wall^6,7^. Conventional NbTi (LTS) shim coils must be placed outside the magnet because of their field limitations: NbTi superconductor is suitable in < 12 T at 1.8 K or < 10 T at 4.2 K. To capitalize on HTS’s ability to operate in fields ≥ 12 T, we introduced in 2013 the first-ever HTS shim coils installable in the cold bore of an NMR magnet of an inside diameter (ID) of < 50 mm^6^: that approach is difficult to scale up for magnet IDs of ≥ 50 mm because of the production limitation of wide (> 46 mm) HTS tape. In continued effort to develop HTS shim coils placeable within the magnet cold bore, we here present another innovative HTS shim design concept readily scalable and thus applicable to UHF NMR magnets such as the MIT 1.3-GHz LTS/HTS high-resolution NMR magnet (1.3G) composed of a 500-MHz LTS NMR magnet (L500) and an 800-MHz HTS insert (H800)^8,9^. We will also apply this HTS shim coil design to our recently proposed all-HTS 1-GHz microcoil NMR magnet (Micro-1G)^10^. For HTS shim coils, we are currently focusing on Z1 and Z2, because not only the SCF in HTS magnets generates large Z1 and Z2 components^5,11^, but also they are simplest to make and install in a limited ≤ 6-mm annular (radial) space available in typical cold bores of HTS magnets, both 1.3G and Micro-1G, as indicated in Fig. 1—note that H835 is a replacement to the original 3-nested-coil 800-MHz HTS insert (H800)^9,12^. The first-step design requirements for HTS Z1 and Z2 shim coils, for 1.3G and Micro-1G, are: (1) maximum strength of ~ 200 kHz/cm^n^, where n = 1 for Z1 and n = 2 for Z2; (2) uniformity of < 1% high-order harmonic errors in 1-cm DSV, i.e., for Z1 of 100 ppm shim strength, < 1 ppm errors in higher odd harmonics: Z3, Z5, … and for Z2, those in higher even harmonics: Z4, Z6, …; (3) minimum B0 field that adds to the main field; (4) minimum inductive coupling with the main magnets; and (5) no overlapping coils to minimize a radial build, i.e., both Z1 and Z2 shim coils to be wound on the same layer. To achieve the ultimate field homogeneity goal for our UHF NMR magnets, 1.3G and Mircro-1G, we will first minimize SCF-induced error fields by our proposed Z1 and Z2 HTS inner shim coils, then apply ferromagnetic shims, which are widely used in conventional LTS NMR^13,14^, HTS NMR^4,5,15,16^ and other UHF NMR magnets^17,18^, and finally rely on room-temperature (RT) copper shim coils^19,20^ for fine tuning.

In this paper, we present the experimental results of a proof-of-concept study of our proposed Z1 and Z2 HTS shim coils obtained with small-scale prototypes. We first show the experimental results of a diamagnetic wall effect with one of the HTS magnets used in H800. Then, we present design, manufacture, and test results of the prototype shim coils. We also discuss error field levels, i.e., required shim strengths, in HTS magnets based on the mapping result of our previous H800, and technical challenges unique in our proposed HTS shim coils.

### Diamagnetic wall effect of HTS magnets

**Figure 2:**
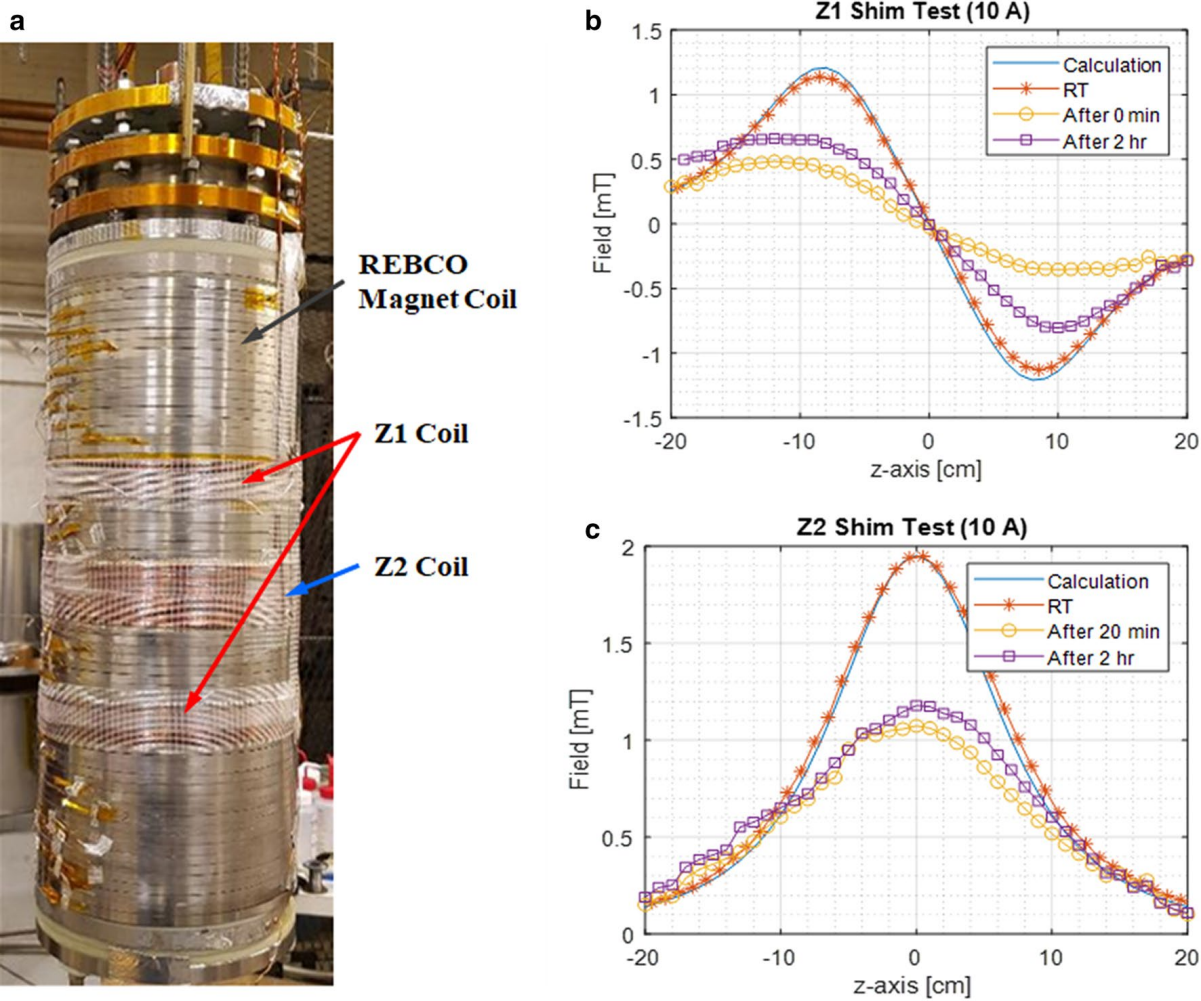
Diamagnetic wall effect test: (**a**) a photo of Z1 and Z2 shim coils wound on top of the REBCO magnet; (**b**) Z1 shim test results; and (**c**) Z2 shim test results.

Diamagnetic wall is a manifestation of a screening current induced by an external field impinging on a cylindrical superconductor^1^. In an ideal “Bean”-tube superconducting cylinder of $\Delta$ thick, a shim field of axial amplitude, generated outside the “Bean” cylinder, is decreased by *J*_*c*_ $\Delta$ where *J*_*c*_ is the superconductor critical current density^1^. Because all conventional LTS magnets are wound with a superconducting wire of < φ100-μm multifilaments, the diamagnetic wall effects are generally negligible. However, most HTS coils, of REBCO or Bi2223, are wound with a tape, monofilament or multifilaments, millimeters wide. Although these sizes are not to be equated to an ideal diamagnetic wall, HTS coils do have attenuating and distorting effects of the diamagnetic walls^21^. We have demonstrated this diamagnetic wall effect in a REBCO magnet for the shim field generated outside the magnet, as shown in Fig. 2a. The REBCO magnet used in this test is a stack of 32 double-pancake coils, 151-mm ID and 169-mm OD, each 120-turn pancake wound with 6-mm-wide, 75-µm-thick REBCO tapes^8^. We wound two shim coils, Z1 and Z2, with φ1-mm copper wires, both placed outside the REBCO magnet and tested them, first the REBCO magnet at room temperature (RT) and then at 77 K in liquid nitrogen bath. Note that the REBCO magnet, superconducting at 77 K, exhibits a diamagnetic effect: Fig. 2b,c show the results of Z1 shim tests and Z2 shim tests, respectively. The measured shim fields along the central axis inside the magnet for both shim coils matched well with the calculation results at RT, but were significantly attenuated and asymmetrically distorted at 77 K, even after two hours as the screening current settled.

### Design, manufacture, and test results of prototype HTS shim coils

**Figure 3:**
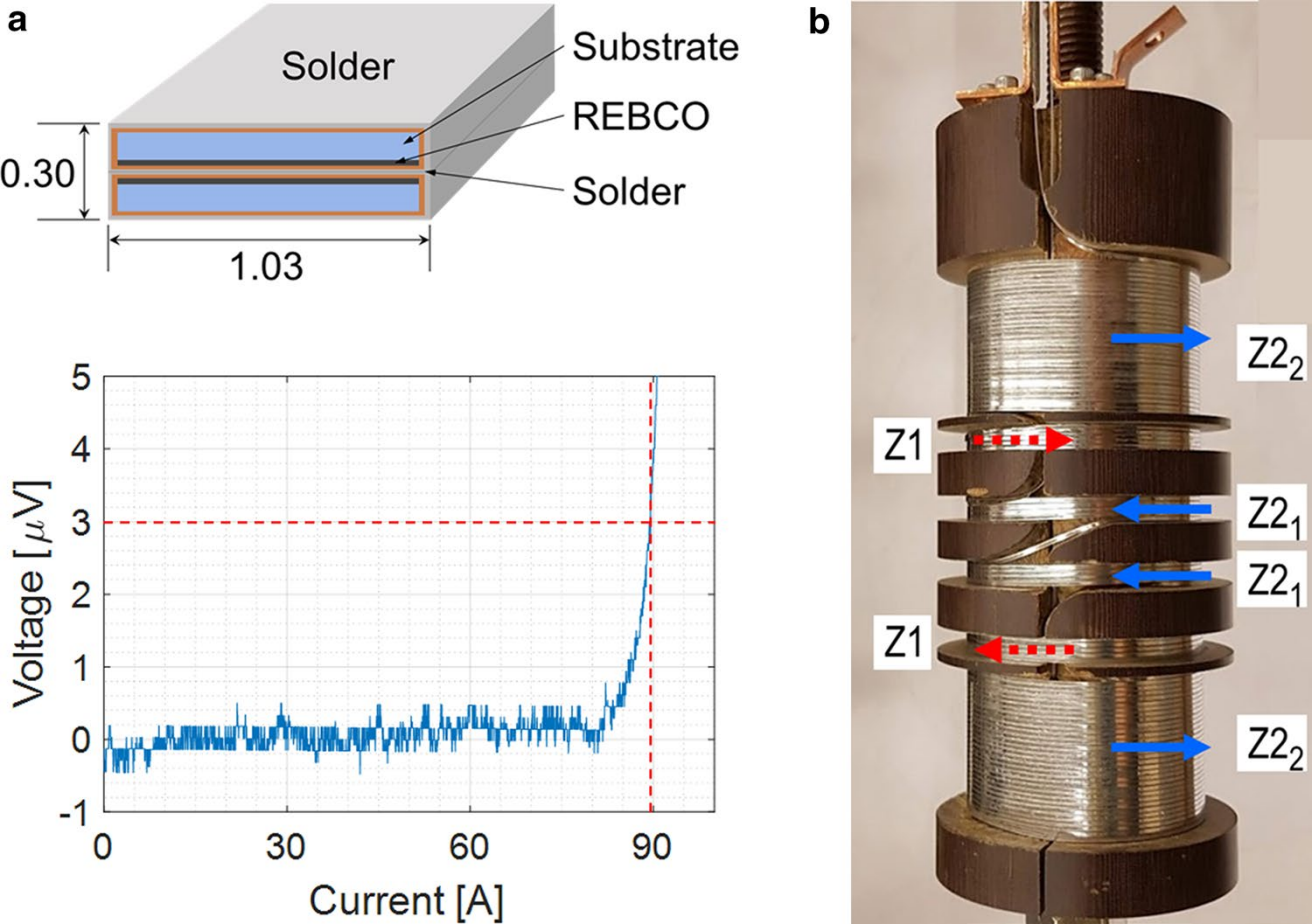
(**a**) Two-pile REBCO conductor cross-section view (dimensions in mm) and *V(I)* plot for 12-cm long sample at 77 K; (**b**) a photo of Z1 and Z2 prototype shim coil sets wound with the 2-pile REBCO conductor. Current directions are denoted with arrows.

We have designed and manufactured two sets of prototype Z1 and Z2 shim coils wound with 2-pile 1.03-mm wide, 0.30-mm thick REBCO conductor. Most REBCO conductors have standard sizes of ≥ 4-mm width and ≤ 0.15-mm thickness, which may not be suitable for our shim coil application because of (1) additional SCF errors from wide REBCO tape; (2) less design freedom than narrower tape conductors; and (3) difficulty of continuous-multi-layer-winding. This unique conductor, developed at Shanghai Jiao Tong University^22^, is a stack of 1-mm wide REBCO tapes slit from a 4-mm wide standard REBCO tape. Two or possibly more 1-mm wide tapes, stacked and soldered, can provide sufficient critical current performance compared with a single 1-mm tape. Figure 3a shows a schematic cross section of this conductor and a *V*(*I*) plot for this 2-pile REBCO conductor sample, 12-cm long, measured at 77 K over voltage taps, 3-cm span. With an *E*-field criterion of 1 μV/cm, the conductor critical current at 77 K in self field is 90 A, sufficient for our Z1 and Z2 shim coil sets operated in a 1.3G field of 30.5 T at 4.2 K and a Micro-1G field of 23.5 T at 10 K, where the principal axial fields are parallel to the conductor broad surface ^23^. Full-scale Z1 and Z2 shim coil sets wound with this type conductor will be designed and installed in 1.3G and similar sets in Micro-1G. For the shim coil application in 1.3G, the operating current is limited to < 80 A because the maximum permissible stress of this 2-pile REBCO conductor is 350 MPa at 4.2 K^22^.

We designed two prototype shim coils, Z1 and Z2, each having a single winding layer of φ50-mm diameter, i.e. on the same layer without crossing each other. We targeted to generate maximum shim strength of ~ 200 kHz/cm^n=1,2^ with ~ 1% high-order errors. The key parameters of the prototype shim coil sets are summarized in Table 1. For these prototype shim sets, one of the shim design requirements, minimum inductive coupling with the main magnet, was overlooked. The Z1 and Z2 shim coil sets require a total conductor length of 13 m. Figure 3b shows a photo of the manufactured Z1 and Z2 prototype shim coil sets, in which current directions are also indicated. A Z2 shim set is composed of two Z2 pairs, Z22 away from the center and Z21 close to the center; Z1 pair is between the Z2 pairs. Each shim coil set was wound with a continuous length (i.e., no joints) of conductor, chiefly to eliminate joule heating at the resistive joint, without turn-to-turn insulation. Contact resistance between side-by-side turns of the 2-pile REBCO conductor is sufficiently high for charging the shim coils without noticeable time delay, characterized by ${L}_{coil}/{R}_{contact}$. These prototype REBCO shim coil sets, as well as full-scale sets for 1.3G and Micro-1G, will operate in driven mode, unlike conventional persistent-mode-operating LTS (NbTi or Nb_3_Sn) shims. This is because a technique to make superconducting joints with REBCO tapes has not been developed; even if it had been, such a technique would unlikely be applicable to this 2-pile REBCO conductor. Full-scale sets in 1.3G and Micro-1G, operated at 4.2 K and 10 K, respectively, will be operated independently but with one common lead, i.e. total three current leads. This is to minimize current-lead heat load to the cold chamber.

**Figure 4:**
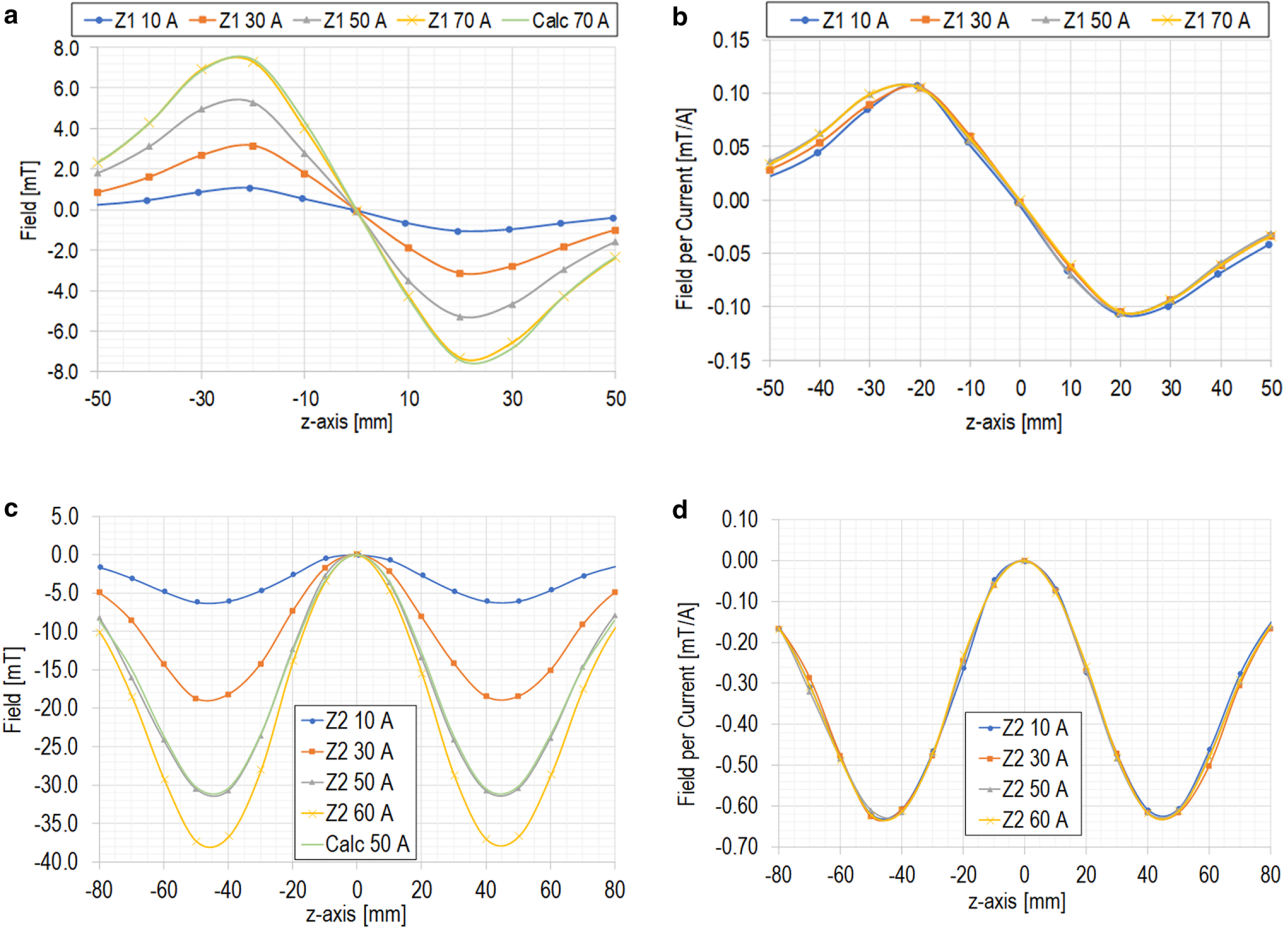
Test results of prototype shim coil sets at 77 K. (**a**) Z1 fields vs. axial position plots, measured at 10, 30, 50, and 70 A, and calculated at 70 A. (**b**) Z1 fields in Fig. 4a per current. (**c**) Z2 fields vs. axial position plots, measured at 10, 30, 50, and 60 A, and calculated at 50 A. (**d**) Z2 fields in Fig. 4c per current.

We tested two prototype shim coils at 77 K in the liquid nitrogen bath. Figure 4 shows (a) Z1 fields vs. axial position plots, measured at 10, 30, 50, and 70 A, and calculated at 70 A; and (b) Z1 fields in Fig. 4a normalized by a unit current. Over nearly a 40-mm along the axial axis, clean Z1 fields are generated. We compute from Fig. 4b a Z1 field of 4.20 mT/cm at 70 A, which, with a proton frequency-field conversion of 42.576 kHz/mT, translates to 179 kHz/cm. This Z1 prototype has a design Z1 parameter of 2674 Hz/cm/A (Table 1), which gives a Z1 field of 187 kHz/cm at 70 A; a ~ 4% disagreement is within experimental uncertainties, mainly of conductor position within the coil form, the conductor geometry, and Hall probe measurement accuracy. Figure 4 also shows (c) Z2 fields vs. axial position plots, measured at 10, 30, 50, and 60 A, and calculated at 50 A; and (d) Z2 fields in Fig. 4c normalized by a unit current. Because of a conductor defect caused during the winding process, we observed a resistive voltage rise in Z22 at above 50 A that forced us to stop at 60 A rather than continued to the maximum design current of 70 A. We compute from Fig. 4b a Z2 field of 3.30 mT/cm^2^ at 50 A, which translates to 141 kHz/cm^2^. This Z2 prototype has a design Z2 parameter of 2965 Hz/cm^2^/A (Table 1), which gives a Z2 field of 148 kHz/cm^2^ at 50 A; a ~ 5% disagreement is again within experimental uncertainties as in Z1. Based on our prototype test results, with further enhanced manufacturing process such as accurately machined bobbin and controlled tension, we strongly believe that the REBCO shim coils are feasible option for our 1.3G and Micro-1G magnet.

**Table 1:** Key parameters of Z1 and Z2 prototype shim coil sets.

Shim coil sets ^a^	Z1	Z2_1_	Z2_2_
*a*_*1*_/*a*_*2*_ [mm]	25.0/25.3		
*b*_*1*_/*b*_*2*_ [mm]	19.3/24.8	4.2/9.7	26.8/60.9
Turns	2 × 5	2 × –5	2 × 31
Total tape length [m]	1.6	11.4	
Z0 [Hz/A]	0.0	2.3	
Z1, or Z2 [Hz/cm^1 or 2^/A]	2674.4	–2965.0	
Z3, or Z4 [Hz/cm^3 or 4^/A]	0.0	19.1	
Z5, or Z6 [Hz/cm^5 or 6^/A]	–33.0	28.2	
Self-inductance [µH]	4.6	84.7	
*I*_*op*_ @ 77 K [A]	≤ 70	≤ 60	

^a^ Field about z = 0: Z1, antisymmetric; Z2, symmetric.

## Discussion on feasibility

**Figure 5:**
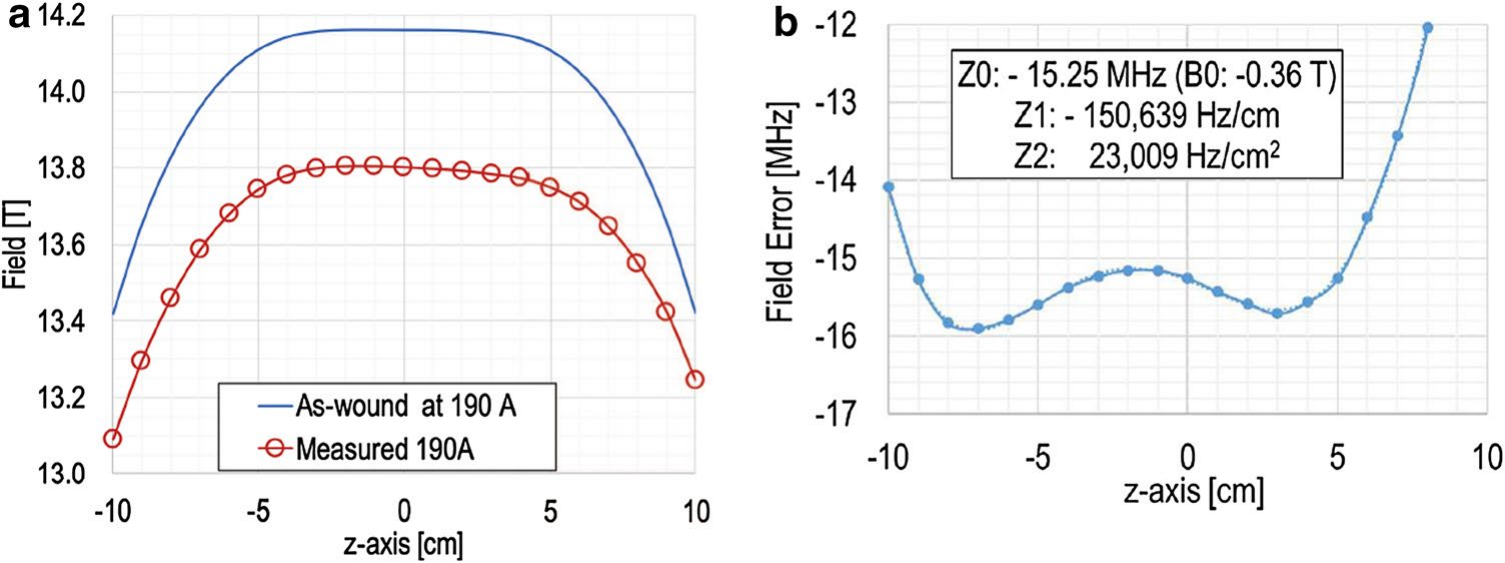
H800 fields vs. axial position plots at 190 A: (**a**) computed (blue line) and measured (red circles and red line) plots; (**b**) The differences in H800 fields, in MHz, between computation and measurement. Z0, Z1, and Z2 error fields given in inset.

Here we discuss the level of field errors produced by our previous 800-MHz REBCO insert (H800) for 1.3G, tested in 2018 to a design operating current, 253.1 A and while it was settling, unexpectedly quenched and then damaged ^12^. Before quench, we measured the axial field plots of H800 at 190 A (13.8 T). Figure 5a shows its computed and measured fields vs. axial position plots at 190 A. The computed field plot is based on actual winding and assembly parameters, e.g., # of turn, ID, outside diameter (OD) of each of 96 double pancake (DP) coils in a 3-nested-coil (Coils 1–3) H800. To make the computed H800 field as symmetric about the magnet center as possible, each coil is placed in the most suitable axial position within H800 assembly. Figure 5b shows the differences in H800 fields, in MHz, between computation and measurement that indicate the extent of total error fields for which H800 is chiefly responsible, its imperfect manufacturing processes and SCF. In the inset, Z0, Z1, and Z2 harmonic field errors computed by polynomial fitting are presented. A 0.36-T center field reduction in the center field is chiefly caused by an SCF induced in 6-mm wide REBCO tape used to wind all 96 DP coils. Measured Z1 and Z2 error terms are, respectively, 151 kHz/cm and 23 kHz/cm^2^. Our prototype Z1 and Z2 sets are able to correct these error fields, except their winding ID of 50 mm is too small for 1.3G which will have a room-temperature bore of 54 mm. Our 1st-cut one-layered design of full scale Z1 and Z2 sets will have an ID of 88 mm and require a ~ 55-m long 2-pile, 1.03-mm wide REBCO tape operating up to 75 A, at which it can generate, with its stand-alone operation, a Z1 shim field of up to 105 kHz/cm and a Z2 shim field of up to 62 kHz/cm^2^. When assembled with a main magnet, even the inner shim coil can be affected by an outside diamagnetic wall, generating lower shim strength than calculation, although it is much less affected than outside shim coils ^5^. As for a Z1 term (< 105 kHz/cm strength vs. 151 kHz/cm error in H800), we may increase the Z1 winding layer from one to two to nearly double a Z1 shim field. Note that a new REBCO insert, H835, and Micro-1G, both in single solenoid configuration, are expected to have a smaller Z1 error field, mostly generated from axial asymmetry, than that in the 3-nested-coil H800. The 1st-cut Z2 shim field can sufficiently compensate 23 kHz/cm^2^ but again the shim strength can be increased by multiple layers, if necessary, still no overlapping with Z1 windings. As for Micro-1G, required ID of Z1 and Z2 sets is 48 mm (Fig. 1) and we will refine our design to minimize the inductive coupling while keeping the shim strength.

The REBCO shim coils will operate in a driven mode, unlike conventional LTS shim operating in a lossless and a very-stable persistent mode. Here, we discuss about two technical issues from a driven mode using a power supply. There will be additional heat loads to a cryogenic chamber (4.2-K liquid helium for 1.3G): conduction through the current leads (up to 75 A); and joule heating from the current leads and resistive joints between coils and leads. We will use HTS current leads to minimize conduction heat loads with nearly zero joule heating. We also measured a joint resistance of ~ 4 µΩ-cm between a 2-pile 1.03-mm REBCO conductor and a 6-mm REBCO tape to be used for an HTS lead. With four 10-cm-long joints (two for each Z1/Z2 shim coil), we expect a total 1.6 µΩ that translates to a maximum total resistive heating at 75 A of ~ 10 mW, a negligible heat load for a > 1-W@4.2-K cryocooler to be installed in 1.3G. Our REBCO shim coils require relatively large-current shim power supplies. As for 1.3G, to meet the NMR required temporal stability of 0.01 ppm/h, the field in 1-cm DSV needs to vary within 13 Hz/h. For example, the maximum field deviation in 1-cm DSV of our 1-cut Z1 shim for 1.3G (105 kHz/cm) is 52.5 kHz at 75 A, and to meet < 13 Hz/h even at the maxim shim strength, the shim field (= current output) variation must be < 250 ppm/h (13/52,500), achievable with modest DC power supplies.

## Conclusion

In this paper, we have presented promising results of prototype REBCO Z1 and Z2 shim sets that we designed, built, and operated. With full-scale version shim sets, specifically targeted to the MIT 1.3-GHz LTS/HTS high-resolution NMR magnet (1.3G) and a tabletop, liquid-helium-free 1-GHz all-HTS microcoil NMR magnet (Mircro-1G), we are confident of minimizing Z1 and Z2 harmonic errors in these UHF HTS NMR magnets as the first and essential step for our ultimate field homogeneity goal, to be finally achieved by following ferromagnetic shims and RT shims. Each full-scale set will be installed in the cold bore of each magnet. It will be the first time that HTS shim coils, placed close to the magnet center, are deployed in UHF NMR magnets.

## References

[1] Iwasa Y (2009). Case Studies in Superconducting Magnet.

[2] Hahn S (2008). Field mapping, NMR lineshape, and screening currents induced field analyses for homogeneity improvement in LTS/HTS NMR magnets. IEEE Trans. Appl. Supercond..

[3] Amemiya N, Akachi K (2008). Magnetic field generated by shielding current in high Tc superconducting coils for NMR magnets. Supercond. Sci. Technol..

[4] Iguchi S (2016). Shimming for the 1020 MHz LTS/Bi-2223 NMR magnet. IEEE Trans. Appl. Supercond..

[5] Iguchi S (2016). Advanced field shimming technology to reduce the influence of a screening current in a REBCO coil for a high-resolution NMR magnet. Supercond. Sci. Technol..

[6] Iwasa Y (2013). Persistent-mode high-temperature superconductor shim coils: A design concept and experimental results of a prototype Z1 high-temperature superconductor shim. Appl. Phys. Lett..

[7] Park D, Lee J, Bascunan J, Michael PC, Iwasa Y (2018). HTS shim coils energized by a flux pump for the MIT 1.3-GHz LTS/HTS NMR magnet: design, construction, and results of a proof-of-concept prototype. IEEE Trans. Appl. Supercond..

[8] Iwasa Y (2015). A high-resolution 1.3-GHz/54-mm LTS/HTS NMR magnet. IEEE Trans. Appl. Supercond..

[9] Park D (2019). MIT 1.3-GHz LTS/HTS NMR magnet: post quench analysis and new 800-MHz insert design. IEEE Trans. Appl. Supercond..

[10] Park D, Choi YH, Iwasa Y (2019). Design of a tabletop liquid-helium-free 23.5-T magnet prototype toward 1-GHz microcoil NMR. IEEE Trans. Appl. Supercond..

[11] Hahn S (2009). Operation and performance analyses of 350 and 700 MHz low-/high-temperature superconductor nuclear magnetic resonance magnets: A march toward operating frequencies above 1 GHz. J. Appl. Phys..

[12] Michael PC (2019). Assembly and test of a 3-nested-coil 800-MHz REBCO Insert (H800) for the MIT 1.3 GHz LTS/HTS NMR magnet. IEEE Trans. Appl. Supercond..

[13] Williams JEC (1992). NMR magnet technology at MIT. IEEE Trans. Magn..

[14] Dorri B, Vermilyea ME, Toffolo WE (1993). Passive shimming of MR magnets: algorithm, hardware, and results. IEEE Trans. Appl. Supercond..

[15] Jang JY (2020). Reproducibility of the field homogeneity of a metal-clad no-insulation all-REBCO magnet with a multi-layer ferromagnetic shim. Supercond. Sci. Technol..

[16] Li, F. X. *et al.* An analytical approach towards passive ferromagnetic shimming design for a high-resolution NMR magnet. *Supercond. Sci. Technol.* **28**, (2015). 10.1088/0953-2048/28/7/075006 PMC4621159 26516300

[17] Gan Z (2017). NMR spectroscopy up to 35.2 T using a series-connected hybrid magnet. J. Magn. Reson..

[18] Litvak IM (2019). Achieving 1 ppm field homogeneity above 24 T: Application of differential mapping for shimming Keck and the Series Connected Hybrid magnets at the NHMFL. J. Magn. Reson..

[19] Sauzade, M. D. & Kan, S. K. High Resolution Nuclear Magnetic Resonance Spectroscopy in High Magnetic Fields. in 1–93 (1973). doi:10.1016/S0065-2539(08)60048-7

[20] Bobrov ES, Punchard WFB (1988). A general method of design of axial and radial shim coils for NMR and MRI magnets. IEEE Trans. Magn..

[21] Hahn S (2008). Development of a 700 MHz low-/high- temperature superconductor nuclear magnetic resonance magnet: test results and spatial homogeneity improvement. Rev. Sci. Instrum..

[22] Li Z (2017). Development of a Novel Soldered-Stacked-Square (3S) HTS wire using 2G narrow tapes with 1 mm width. IEEE Trans. Appl. Supercond..

[23] Tsuchiya K (2017). Critical current measurement of commercial REBCO conductors at 4.2 K. Cryogenics (Guildf)..

