# Publisher Correction: Prototype REBCO Z1 and Z2 shim coils for ultra high-field high-temperature superconducting NMR magnets

**DOI:** 10.1038/s41598-021-87437-y

**Published:** 2021-04-15

**Authors:** Dongkeun Park, Jiho Lee, Juan Bascuñán, Zhuyong Li, Yukikazu Iwasa

**Affiliations:** 1 Francis Bitter Magnet Laboratory/Plasma Science and Fusion Center, Massachusetts Institute of Technology, Cambridge, MA 02139 USA; 2 Institute for Basic Science, Daejeon, 34126 South Korea; 3 Department of Electrical Engineering, Shanghai Jiao Tong University, Shanghai, 200240 China

Correction to: *Scientific Reports* 10.1038/s41598-020-78644-0, published online 15 December 2020

In the original version of this Article part of the title was erroneously omitted.

“Prototype REBCO Z1 and Z2 shim coils for ultra high-field”

now reads:

“Prototype REBCO Z1 and Z2 shim coils for ultra high-field high-temperature superconducting NMR magnets”

The original Article has been corrected.

